# A systematic review of the association between parastomal hernia and sarcopenia

**DOI:** 10.1007/s00384-023-04329-5

**Published:** 2023-02-09

**Authors:** Grant Schutte, Declan Patton, Zena Moore, Deborah McNamara, Tom O’Connor, Linda Nugent, Pinar Avsar

**Affiliations:** 1 School of Medicine, Royal College of Surgeons in Ireland University of Medicine and Health Sciences, Dublin, Ireland; 2 Skin Wounds and Trauma Research Centre, RCSI University of Medicine and Health Sciences, Dublin, Ireland; 3 School of Nursing and Midwifery, RCSI University of Medicine and Health Sciences, Dublin, Ireland; 4 Fakeeh College of Health Sciences, Jeddah, Saudi Arabia; 5 School of Nursing and Midwifery, Griffith University, Queensland, Australia; 6 Faculty of Science, Medicine and Health, University of Wollongong, Wollongong, Australia; 7 Faculty of Medicine, Nursing and Health Sciences, Monash University, Melbourne, Australia; 8 Department of Public Health, Faculty of Medicine and Health Sciences, Ghent University, Ghent, Belgium; 9 Lida Institute, Shanghai, China; 10 University of Wales, Cardiff, UK; 11 National Health and Medical Research Council Centre of Research Excellence in Wiser Wound Care, Menzies Health Institute Queensland, Southport, QLD Australia; 12 Beaumont Hospital, Dublin, Ireland; 13 RCSI University of Medicine and Health Sciences, Dublin, Ireland

**Keywords:** Sarcopenia, Parastomal hernia, Hernia

## Abstract

**Background:**

Sarcopenia is a multifactorial loss of muscle mass that can complicate surgical outcomes and increase morbidity and mortality. Parastomal hernias can occur after any surgery requiring stoma formation and is an area of concern as a complication as it can require a second surgery or emergency surgical intervention.

**Aim:**

To assess the impact of sarcopenia on parastomal hernia formation in the postoperative period.

**Method:**

A systematic search of publications using MEDLINE, CINAHL, and Cochrane databases was conducted in June 2022. Data were extracted, and a narrative synthesis was undertaken. The Crowe Critical Appraisal Tool (CCAT) assessed the quality of the included studies. The systematic review included original research studies, prospective and retrospective designs, and human studies written in English*.* Reviews, conference papers, opinion papers, and those including participants < 18 years old were excluded. No restrictions on the date of publication and study setting were applied.

**Results:**

Nine studies met the inclusion criteria, and these were conducted between 2016 and 2021; 56% (*n* = 5) used a retrospective study design. The mean sample size was 242.5 participants (SD = ±358.6). No consistent or standardized way of defining sarcopenia or measuring muscle mass was seen between the studies reviewed. However, 45% (*n* = 4) of the studies reported a significant relationship between sarcopenia and wound healing complications, including an increased incidence of parastomal and incisional hernias. The average CCAT score was 27.56 (SD = ±4.39).

**Conclusion:**

There is no definitive relationship between sarcopenia and hernia development; however, four studies found a significant relationship between sarcopenia and hernia formation. It must also be considered that different disease processes can cause sarcopenia either through the disease process itself, or the treatment and management. More research and consistent measurements are needed before comparable and consistent outcomes can be compiled.

## Introduction

Sarcopenia is when an individual’s muscle mass decreases compared to others of the same age, race, and gender [1]. It is a multifactorial process that can result from chronic illness, cancer, lack of activity, or a combination of these and many more factors [2–5]. Sarcopenia is not solely a disease of the old and frail but is seen in patients with increased BMIs and is independent of age [1, 3, 4]. This loss of muscle mass not only has an impact on activities of daily life but is a known risk factor for postoperative morbidity and mortality in both the immediate and long-term postoperative periods [2–4, 6–10]. Many theories, such as chronically increased inflammatory processes, or a state of increased long-term catabolism, have been posited as reasons for the worsened postoperative outcomes, but no definitive etiology has been found [3, 6, 10]. The postoperative complications from sarcopenia are commonly but not limited to reduced survival, increased length of stay, higher rates of sepsis, a need for reoperations, increased need for rehabilitation, impaired physical capability, and decreased ability to cope with stressors, all of which increase cost and demand on the health care system [2, 3, 6, 8, 10, 11].

A parastomal hernia (PSH) occurs when a portion of the abdominal organs protrudes through the incision created for or around the stoma [12], in other words, an incisional hernia at the site of an abdominal wall ostomy. A variety of stomas are created for different indications including loop and end ileostomy and loop or end colostomy. The incidence of PSH varies between types of stoma and with technique of follow-up [7, 12–14]. The incidence increases with time, a factor that is important since the stoma may be long-term or permanent for many patients. Almost one in three patients will report a PSH within a year and reach 50% over 2 years, with some studies reporting that up to 80% of patients will develop a PSH [7, 12–14]. A PSH may present asymptomatically, but emergency surgery is required in some cases due to incarceration or obstruction [12–14]. While there is no consensus on grading a PSH, the European Hernia Society has created the most recent guidelines [15]. They grade the PSH based on the size and presence of concomitant incisional hernias giving some guidance on the classification of PSHs.

Interestingly, the literature focuses to a much greater extent on the presence of obesity than the absence of muscle mass as a causative factor for PSH. Clinically, sarcopenia is a potentially reversible cause of postoperative complications and can be treated with nutritional support or physical therapy combined with nutrition and exercise [1, 9, 11]. Sahebally et al. showed that increased abdominal wall fat had positive postoperative outcomes in laparotomy patients whereas obesity is associated with an increased risk of parastomal hernia [16]. Chronically sick patients are less active and older in general, and their sarcopenia may be present at the time of cancer diagnosis [2, 3, 10]. Identifying and creating a prophylactic nutritional or rehabilitation program for a patient population that is chronically ill and older is not without its challenges but may be of benefit to their overall health status. Clearer understanding of the relationship between sarcopenia and PSH may allow surgical mitigation using specific surgical strategies, such as mesh insertion at the time of surgery. To date, there are no studies looking at the link between PSH and sarcopenia. This review aims to synthesize available literature on the incidence of sarcopenia and patients with parastomal hernias, what patient-related risk factors are present, and the impact of these complications.

## Methods

### Aim and research question

The aim of the systematic review was to explore the association between parastomal hernia and sarcopenia. The research question was: “What is the association between parastomal hernia and sarcopenia?”.

### Outcomes measured

The primary objective was to explore the association between parastomal hernia and sarcopenia. The secondary objective was to determine the impact of parastomal hernia and sarcopenia on quality of life, length of stay, and mortality.

### Search strategy

We included various research designs such as randomized controlled trials (RCT), case–control studies, and observational cohort studies written in English. Reviews, conference papers, opinion papers, and those including participants < 18 years old were excluded. No restrictions on the date of publication and study setting were applied. Searches were conducted on a variety of databases outlined below using a set of pre-determined keywords. Abstracts and titles were then screened for eligibility by two authors (GS, PA).

#### Inclusion criteria: quantitative study designs that report the association between parastomal hernia and sarcopenia

Varied research designs such as randomized controlled trials (RCT), case–control studies, and observational cohort studies can be anticipated in order to assess and observe risk factors within this cohort.

#### Exclusion criteria

Reviews, conference papers, opinion papers, and those including participants < 18 years old were excluded.

### Electronic searches

The following databases were searched to identify relevant literature:

- Cochrane Central Register of Controlled Trials (CENTRAL) (The Cochrane Library) (latest issue);
- Ovid MEDLINE (1946 to April 2022);
- Ovid MEDLINE (in-process and other non-indexed citations) (latest issue); and
- EBSCO CINAHL Plus (1937 to April 2022).

Key terms, MeSH terms and subject heading were used. The literature was reviewed by one of the authors, and selected for inclusion in this study according to the eligibility criteria outlined above.

Terms used for each database:

(“sarcopenia” OR “core muscle” OR “body composition” OR “myopenia”) AND (“abdominal wall reconstruction” OR “ventral hernia repair” OR “hernia” OR “complex abdominal wall” “parastomal” OR “stomal” OR “incisional”).

To identify further published, unpublished, and ongoing studies, we do the following:

- Scanned reference lists of all identified studies and reviews to assess for further relevant citations;
- Performed a manual search of relevant grey literature, to enhance the capture of relevant and unique literature (i.e. OpenGrey www.opengrey.eu); and
- Conducted searches of conference proceedings, research reports and dissertations.

### Study selection

Articles deemed relevant were then examined in full text by two authors (GS, PA) for further analysis. The references list of each was also screened for further potential eligible articles. The final list of articles was then agreed on and approved by all authors (DP, ZM, DM) prior to data extraction.

### Data extraction

Data were extracted using an approved data extraction table, by (GS) and included study design, interventions, sample size, characteristics, method of evaluation, key findings, and limitations. Data entry was checked by a second reviewer (PA).

### Data analysis

Following data extraction, meta-analysis statistical synthesis was considered inappropriate. Thus, first, the data were narratively summarized, giving an overview of the study setting, geographical location, study settings, sample sizes, and primary and secondary outcomes. This was followed by quality appraisal and a structured narrative synthesis of all the outcomes of the studies included. The studies were quality appraised using Crowe Critical Appraisal Tool (CCAT) version 1.4 [17]. This tool was selected as it was anticipated that the studies included would have significantly different methodologies. The CCAT is divided into eight categories and 22 items. Each item has multiple descriptors for ease of appraisal, with each category receiving its own score on a 6-point scale (0–5). An overall score for each study can be expressed out of a total score of 40 points. Two independent raters assessed each study. Discrepancies were resolved after a discussion between the authors.

## Result

### Overview of all included studies

**Fig. 1:**
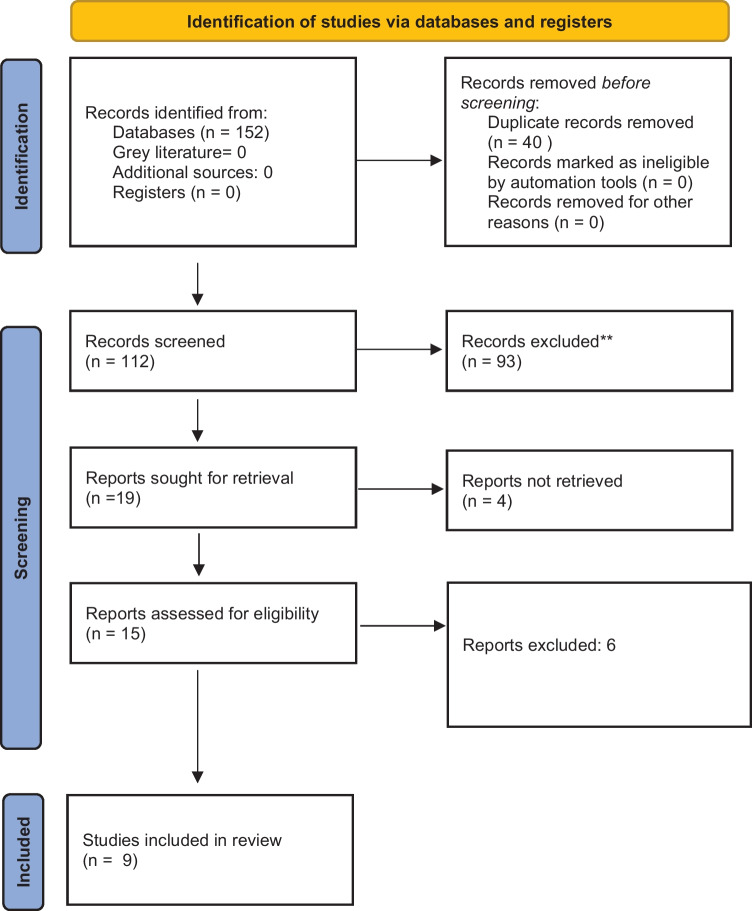
PRISMA flow diagram for study selection [32]

Figure 1 outlines the flow of articles through the reviews. As can be seen, following reviews of titles and abstracts from a total of 152 hits, 137 were excluded. Four studies were excluded because the abstract was only available and the full study was not retrievable in any database. Then, following a review of the full papers of the remaining hits, six were rejected for the following reasons: did not measure stoma incidence or complications with stomas [18], the study only looked at inguinal hernias [19], sarcopenia was not directly measured [20], surgery was only for colonic perforation [21], not all patients undergoing surgery received a stoma [4], and did not explore the complications of stomas [22] (see Table 1). Thus nine studies were finally included [23–31] and form the basis of this review.

**Table 1:** Excluded studies with reasons

**Author**	**Reason for exclusion**
Zhang et al. (2020)	Did not measure stoma incidence or complications with stomas
Umeda et al. (2022)	Only studied inguinal hernias
Pennings et al. (2021)	No measurement of sarcopenia
Imamura et al. (2019)	Surgery for colonic perforation
Zhang et al. (2017)	Not all patients received a stoma
Huang et al. (2015)	Does not explore the complications of stomas

### Study design

Of the included studies, one employed a cross-sectional design [25]. Five authors employed a retrospective study design [23, 24, 26–28]. Two used prospective designs, [29, 31], and one was a randomized control trial [30] (see Table 2).

**Table 2:** Study characteristic

**Authors and country**	**Design**	**Study setting**	**Study population**	**Surgery performed**	**Hernia characteristics**
Bailey et al. (2020), USA	Retrospective cohort	University cancer center	86 patients	AWR for an ablative abdominal wall defect	Ventral hernia working group grade
Barnes et al. (2018), USA	Retrospective cohort	University medical center	58 patients	Ventral hernia repair	Hernia transverse size cm^2^
Du et al. (2021), China	Cross sectional	Hospital	120 patients	Appendectomy	
Ishimaru et al. (2021), Japan	Retrospective cohort	University medical center	134 patients	Loop ileostomy or loop colostomy via the intraperitoneal route	Devlin classified parastomal hernia: interstitial, subcutaneous, intrastomal, and peristomal
Ki et al. (2020) Korea	Retrospective cohort	University medical center	18 patients	Laparoscopic abdominal surgery	Tonouchi 2004 classification system for PSH in 2004, (1) early-onset type: dehiscence of the anterior and posterior fascial plane and peritoneum, (2) late-onset type: dehiscence of the anterior and posterior fascial plane. Peritoneum constitutes hernia sac, (3) special type: dehiscence of the whole abdominal wall, the protrusion on intestine and/or omentum
Otaki et al. (2021), Japan	Retrospective cohort	University medical center	147 patients	Robot-assisted laparoscopic radical prostatectomy	
Rinaldi et al. (2016), USA	Prospective cohort	University medical center	159 patients	Hernia repair	Hernia defect area, hernia volume
Schlosser et al. (2019), USA	Prospective cohort	Regional medical center	1178 patients	Open ventral hernia repair	Location, defect size, volume
van Rooijen et al. (2019), Netherlands	Randomized control trial	Multicenter	545 patients	Elective midline laparotomy	

### Geographical location

The geographical location of the studies varied between the United States [23, 24, 29, 31], Japan [26, 28], China [25], the Netherlands [30] and Korea [27] (see Table 2).

### Study settings

The study settings varied and included a university cancer center [23], a hospital [25], a university medical center [24, 26–29] a regional medical center [31], and a multicenter trial [30] (see Table 1). As can be seen, the university medical center setting was the most common study site, accounting for 56% (*n* = 5) of all the other care settings, and the lowest rate (11%; *n* = 1) was reported from a university cancer center, a regional medical center, and a hospital setting (see Table 2).

### Sample size

The mean sample size was 242.5 participants (SD = ±358.6), varying between 18 participants [27] and 1178 participants [31]. There were 2183 patients enrolled in the nine studies (see Table 2).

### Population

In all nine studies, the participants were all undergoing surgery (see Table 2).

### Study period

All nine studies were conducted between 2 and 11 years with an average of 5.2 years (SD = ±3.5).

### Hernia characteristic

There were many different ways the studies reported hernia characteristics. Many studies used predefined criteria; Bailey et al. [23] used the ventral working group grade, Ishimaru et al. [26], used the Devlin classified parastomal hernia criteria, and Ki et al. [27] used Tonouchi et al. [33] classification system for PSH. Others used measurements of the hernias; Barnes et al. [24] used transverse hernia size, Rinaldi et al. [29] used hernia defect area and hernia volume, and Schlosser et al. [31] reported hernia location, size, and volume. The rest of the studies did not include measurements or descriptions of the hernias observed [25, 28, 30].

### Results for the primary outcome

Study outcomes are reported in Table 3. Six studies analyzed postoperative complications [23, 24, 26, 27, 30, 31]. In contrast, three others looked at pre and postoperative factors, including patient characteristics that could influence hernia formation and rates of sarcopenia [25, 28, 29]. Four studies found associations between sarcopenia and hernia formation [24–26, 28].

**Table 3:** Primary and secondary outcomes for included studies

**Study**	**Core muscle analysis**	**Primary outcome result**	**Secondary outcome result**
Bailey et al. (2020)	Total psoas index at L3	• Hernia occurrence Sarcopenia not significant (*p* = 0.12)	• SSO: sarcopenia not a significant factor (*p* = 0.85)
Barnes et al. (2018)	Cross-sectional psoas muscle area L4	• Delayed wound healing and surgical site/mesh infection more likely with sarcopenia (*p* = 0.04)	• Incidence of hernia recurrence increased and renal failure with sarcopenia (*p* = 0.04, *p* = 0.03)
Du et al. (2021)	PMI L3	• Demographic comparisons for psoas muscle index in the incisional hernia group were significantly lower than those in the non-incision hernia group (*Z* = − 2.03, *p* = 0.042) as well as CT attenuation (*t* = − 2.18,* p* = 0.031) • The fatty infiltration rate in incisional hernia patients was statistically higher than in non-incisional hernia patients (*Z* = 3.57, *p* < 0.001)	• CT attenuation of abdominal wall muscle an independent protective factor (OR 0.94, 95% CI 0.88–0.99, *p* = 0.042) • Fatty infiltration rate—an independent risk factor (OR 1.34, 95% CI 1.05–1.70, *p* = 0.018) • Psoas muscle index and sarcopenia showed no effect on incisional hernias (OR 0.78, 95% CI 0.56–1.07, *p* = 0.118), (OR 0.84, 95% CI 0.38–1.87, *p* = 0.663)
Ishimaru et al. (2021)	PMI L3	• Incidence of parastomal hernia increased with a diagnosis of sarcopenia (*p* = 0.018)	• Sarcopenia increased the risk of parastomal hernia on multivariate analysis (OR, 5.08; 95% CI, 1.10–25.8; *p* = 0.039)
Ki et al. (2020)	PMI L3	• Diastasis recti and port site hernia not significant (*p* = 0.263)	• Relationship of risk factors for port site hernia showed nine (50%) patients with sarcopenia
Otaki et al. (2021)	PMV – total TRM – umbilical	• PMV < 350 cm^3^— a significant independent risk factor for postoperative inguinal hernias (*p* = 0.03; HR, 2.19; 95% CI, 1.07–4.50)	• The postoperative inguinal hernias-free rate at 1, 2, and 3 years postoperatively was 91.5%, 83.4%, and 83.4% in patients with a PMV > 350 cm^3^, and 77.4%, 68.9%, and 58.4% in patients with a PMV < 350 cm^3^ (*p* = 0.01)
Rinaldi et al. (2016)	SMI L3	• Prevalence of sarcopenia and sarcopenic obesity in this patient population. Sarcopenia was found in 38 (26%) of the 148 patients and 29 (23%) of the 127 patients with obesity	• Surgical site outcomes and reoccurrence not associated with sarcopenia (*p* = 0.1137, *p* = 1.000) • Length of stay and duration of ileus associated with sarcopenic patients (*p* = 0.0218, *p* = 0.0156), and sarcopenic-obese patients (*p* = 0.0117, *p* = 0.0025) • Surgical site occurrence and reoccurrence not significant (*p* = 0.7429, *p* = 0.7061) • Positive associations were observed for SMI with serum hemoglobin and alanine ALT (Pearson *r* = 0.31, *p* = 0.003, *r* = 0.19, 0.0486)
Schlosser et al. (2019)	SMI L3	• Wound complications, hernia recurrence, and major complications of Clavien-Dindo grade 3 or greater are not significant to sarcopenia (*p* = 0.7, 0.8, 0.2) • Osteopenia nor sarcopenia were found to be associated with wound complications, major complications, reoperation, readmission, or hernia recurrence	• The relationship of sarcopenia and reoperation (*p* = 0.2), length of stay, operative time, and readmission (*p* = 0.32)
van Rooijen et al. (2019)	SMI L3	• The development of an incisional hernia Nagelkerke’s *R*^2^, sarcopenia has a 1.0–2.7% share in the variation in occurrence of incisional hernia	• 18.8% of patients with sarcopenia developed a hernia, compared to 18.6% without sarcopenia • When sarcopenia was measured through cutoff values from the literature, 19.9% of patients with sarcopenia developed an hernia, compared to 17.3% without sarcopenia

Barnes et al. [24] showed that the rate of wound complications was five times more likely in patients with sarcopenia (OR = 5.313, CI 1.121–25174, *p* = 0.04) and was statistically significantly different overall between sarcopenic and nonsarcopenic patients (*p* = 0.03). This included delayed healing and surgical site and mesh site infections.

Du et al. [25] compared the patient characteristics for incisional hernias using abdominal muscle CT attenuation, psoas muscle index, and fatty infiltration rate. The authors found that the patients with low CT attenuation of abdominal muscles were more likely to have an incisional hernia (*t* = −2.18, *p* = 0.031), have a greater fatty infiltration rate (*Z* = 3.57, *p* = 0.001), and have a lower psoas muscle index (*Z* = −2.03, *p* = 0.042). This shows that not only does decrease muscle mass impact hernias, shown as CT attenuation and psoas muscle index, but also the quality of the muscle itself via the fatty infiltration rate.

Ishimaru et al. [26] studied parastomal hernias and sarcopenia directly. In their cohort, there was a higher incidence of sarcopenia in the group with parastomal hernias (*p* = 0.018).

Otaki et al. [28] identified low psoas muscle volume as more common in their hernia-positive group (*p* < 0.05) and a statistically significant independent risk factor for umbilical incisional hernia with a hazard ratio of 2.19 (95% CI, 1.07–4.50 *p* = 0.03).

Bailey et al. [23] found no relationship between sarcopenia and hernia occurrence (*p* = 0.12) on physical examinations or postoperative CT surveillance scans.

Ki et al. [27] used diastasis recti and psoas muscle index as markers of sarcopenia for port site hernias. They found no statistically significant relationship between diastasis recti and hernias (*p* = 0.263).

Schlosser et al. [31] explored sarcopenia and ventral hernia repair. They found no association between wound complications, hernia recurrence, and major complications of Clavien-Dindo grade 3 or greater with sarcopenia (*p* = 0.7; *p* = 0.8; *p* = 0.2). Neither osteopenia nor sarcopenia was associated with wound complications, major complications, reoperation, readmission, or hernia recurrence.

van Rooijen et al. [30] used the STITCH randomized control surgical trial to investigate sarcopenia and its predictive value to incisional hernias. The authors looked at the lowest gender-specific quartile and measurements set by Martin et al. [35]. They found no statistically significant relationship between sarcopenia and hernia development or between sarcopenia and other postoperative outcomes despite many different models and cutoff values. The primary outcome measure for the study was the development of an incisional hernia Nagelkerke’s *R*^2^, sarcopenia has a 1.0–2.7% share in the variation in the occurrence of incisional hernia.

Rinaldi et al. [29] reported on their use of CT measurements to measure the prevalence of sarcopenia and sarcopenic-obese patients. They found that 26% (38 of 148) of their cohort had sarcopenia. In the 127 patients with obesity (BMI > 24.9 kg/m^2^), they found that 23% (29/127) concurrently had sarcopenia.

### Results for the secondary outcomes

Secondary study outcomes are reported in Table 3. The secondary outcomes varied in each study. Analysis ranged from logistic and multivariate analyses [25, 26] and exploration of the relationship between complications and incidence reporting [23, 24, 27–31].

Bailey et al. [23] found that sarcopenia and surgical site occurrence (which was defined by an infection requiring antibiotics, drainage, fat necrosis over 1 cm persisting for more than 3 months, and wound dehiscence greater than 2 cm requiring intervention) had no statistically significant relationship (*p* = 0.85).

Barnes et al. [24] studied the incidence of hernia recurrence in their patient population. The authors found that it was increased with sarcopenia, with 7 (33%) of sarcopenic patients having an hernia reoccurrence vs 4 (11%) without sarcopenia (*p* = 0.03).

Du et al. [25] ran a univariate logistic regression analysis that investigated the psoas’ CT attenuation, fatty infiltration rate, and psoas muscle index PMI as independent factors associated with an incisional hernia. CT attenuation was deemed an independent protective factor (OR 0.94, 95% CI 0.88–0.99, *p* = 0.042), and the fatty infiltration rate was an independent risk factor (OR 1.34, 95% CI 1.05–1.70, *p* = 0.018). In contrast, psoas muscle index had no effect on incisional hernias (OR 0.78, 95% CI 0.56–1.07, *p* = 0.118) and neither did sarcopenia (OR 0.84, 95% CI 0.38–1.87, *p* = 0.663).

Ishimaru et al. [26] ran a multivariate analysis that showed that sarcopenia increases the risk of parastomal hernia by five times (OR 5.08; 95% CI, 1.10–25.8; *p* = 0.039).

In Ki et al. [27], the authors were able to report but did not statistically analyze sarcopenia and hernias. However, they found that 50% of their hernia group had sarcopenia, and 60% of the diastasis recti group had sarcopenia on CT.

Otaki et al. [28] followed up with their participants over 3 years. The postoperative inguinal hernias-free rate at 1, 2, and 3 years postoperatively was 91.5%, 83.4%, and 83.4% among patients without sarcopenia, and 77.4%, 68.9%, and 58.4% among patients with sarcopenia (*p* = 0.01).

Rinaldi et al. [29] found that surgical site outcomes and reoccurrence were not statistically significantly associated with sarcopenia (*p* = 0.1137, *p* = 1.000). Surgical site occurrence and reoccurrence were not statistically significant in sarcopenic and sarcopenic-obese patients (*p* = 0.7429, *p* = 0.7061). Duration of ileus and length of stay was statistically significant in sarcopenic and sarcopenic-obese patients (*p* = 0.0117, *p* = 0.0025). Positive associations were observed for skeletal muscle index with serum hemoglobin and alanine ALT (Pearson *r* = 0.31, *p* = 0.003, *r* = 0.19, 0.0486).

In Schlosser et al. [31], their secondary outcomes included reoperation (*p* = 0.2), length of stay, operative time, and readmission (*p* = 0.32) with no statistically significant relationship with sarcopenia identified.

van Rooijen et al. [30] showed that 18.8% of people with sarcopenia developed an hernia, compared to 18.6% of people without sarcopenia when defined by the lowest gender-specific cutoffs. When sarcopenia was measured through cutoff values from the literature of Martin et al. [35], 19.9% of people with sarcopenia developed an hernia, compared to 17.3% of people without sarcopenia. The authors show that their results point towards the absence of predictive value of sarcopenia for developing postoperative complications.

### Quality appraisal

Two raters appraised each of the nine studies, which resulted in nine independent CCAT evaluations; the total score ranged from 19 to 34 out of 40 (see Table 4). The overall assessment mean for all studies was 27.56 out of 40 points (moderate scoring), with a standard deviation of 4.39. Within the CCAT sections, the highest scores were for preliminary (4.22/5) and discussion (4.00/5), while the lowest was for ethics (2.45/5) and results (3.11/5).

**Table 4:** Quality appraisal using the CCAT

**Study**	**Preliminaries**	**Introduction**	**Design**	**Sampling**	**Data collection**	**Ethical matters**	**Results**	**Discussion**	**Total**
Bailey et al. (2020)	5	4	4	4	5	3	4	5	34
Barnes et al. (2018)	5	5	3	4	3	3	4	5	32
Du et al. (2021)	4	3	3	3	3	2	2	4	24
Ishimaru et al. (2021)	4	3	3	4	3	3	4	4	28
Ki et al. (2020)	4	2	3	2	2	2	2	2	19
Otaki et al. (2021)	4	4	3	3	4	2	3	4	27
Rinaldi et al. (2016)	5	4	3	4	4	2	3	4	29
Schlosser et al. (2019)	3	4	4	3	3	2	3	4	26
van Rooijen et al. (2019)	4	4	4	4	3	3	3	4	29
Average (SD)	4.22 (0.67)	3.67 (0.87)	3.33 (0.50)	3.44 (0.73)	3.33 (0.87)	2.44 (0.53)	3.11 (0.78)	4.00 (0.87)	27.56 (4.39)

## Discussion

Sarcopenia is a significant and potentially modifiable factor in hernia development. With an aging population and a reported prevalence varying from 10 to 67%, sarcopenia should be considered a modifiable patient factor in the preparation for surgery [31, 34]. Without consensus on the cutoff value for sarcopenia and a standardized way to measure muscle mass, comparison of data between studies is challenging. Of the nine studies reviewed, three used the skeletal muscle index (SMI) at the third lumbar vertebral level (L3) [29–31], while others used the psoas muscle index (PMI) at L3 [25–27], the total psoas muscle volume (PMV) [28], the total psoas index (TPI) at L3 [23], and a combination of the cross-sectional psoas muscle area and the fourth lumbar vertebral level (L4) [24]. The definition of sarcopenia also varied between studies. The lowest sex-specific quartile was used by [23, 30] with additional measurements that were set by [35] and utilized by [30]. Gender-specific cutoff points were used by [31]. In the four studies set in Asia [25–28], cutoffs based on the Asian population were appropriately used, but these varied with [26, 27] using measurement cutoffs from [36] and [25] used values from [37]. Rinaldi et al. [29], had gender-specific cutoffs, but these differed from the other studies. Finally, both Barnes et al. [24] and Otaki et al. [28], used more arbitrary cutoff values based on their patient populations. Given this heterogeneity in the definition of sarcopenia, the lack of consensus achieved among reviewed studies is not surprising.

Despite this limitation, a number of trends may be observed. Five studies reported a statistically significant relationship between sarcopenia and wound healing complications and an increased incidence of parastomal and incisional hernias [24–26, 28, 29]. Incisional hernias are the most common complication of laparotomy, occurring in approximately 11–30% of cases [38]., with five studies showing that sarcopenia is related to hernia formation [24–26, 28, 29]. Not only does the lack of muscle, as defined by sarcopenia, tend to lead to poorer outcomes with stoma and hernias, but the quality of the muscle is also a critical factor [25, 30]. The fatty infiltration rate of the psoas muscle was an independent predictive factor for incisional hernia development [25]. Sarcopenic-obese patients also had poorer outcomes. Increased hospital costs are associated with sarcopenia because the length of hospital stay and duration of ileus are increased [30]. More prolonged admissions may lead to an increased risk of nosocomial infections and other complications associated with long-term hospital stays.

This review has important limitations. First, the overall quality of the evidence base is moderate at best, scoring 27.56 out of 40 on the CCAT scale, mainly due to poor reporting of results and ethics approval. Although poor ranking in the results and ethics disclosure ranking may not introduce bias, it may raise concern regarding the rigor of study design and execution. All included studies were published in peer-reviewed journals, a majority of which require ethical disclosure prior to publication, but sufficient information was absent in the final published report. Second, as previously noted, evaluation of sarcopenia lacks standardization. Finally, the variability between studies is significant with patient populations, hospital context, and procedures performed heterogeneous in all regards. Taken together, these limitations make clear that, furthermore, rigorously conducted studies are needed to advance our understanding of this clinically important problem. Prehabilitation that includes physical exercise and nutritional optimization improves surgical outcomes [39, 40], and its impact on the incidence of PSH should be evaluated. Standardized measurement of preoperative muscle mass should be mandatory in future studies on incisional and parastomal hernia to properly assess whether technical interventions, like prophylactic mesh insertion, have differing risk/benefit profiles in sarcopenic patients. In an era of personalized medicine, proposed study of the complex relationship between sarcopenia, obesity and metabolic syndrome, and surgical outcomes is welcome [41]. A combination of lifestyle medicine and surgical mitigation strategies may have potential to target patients at highest risk and reduce the burden of PSH on patients and the healthcare system.

## Conclusion

Sarcopenia continues to be a factor to contend within the surgical patient population. While there is no definitive relationship between sarcopenia and hernia development, there are some trends that are worth considering in patients. It might also be considered that different disease processes can cause sarcopenia either through the disease process or the treatment and management. This systematic review was designed to determine the association between parastomal hernia and sarcopenia. In nine studies, there were five different measurements of sarcopenia with eight different cutoff values. The lack of consensus on sarcopenia measurements hinders the scientific communities’ ability to correctly identify and appropriately prepare management for their specific patient population. Preoperative management and treatment of sarcopenia have shown promise, and if a strong and reliable relationship can be shown between sarcopenia and hernia formation, there may be a way to prevent hernias and other poor surgical outcomes from sarcopenia altogether.

## Data Availability

Data sharing not applicable to this article as it is a systematic review.
